# Root Cause Analysis of Gaps in Non-communicable Disease Monitoring in a Sub-district Hospital, Tamil Nadu: A Quality Improvement Initiative

**DOI:** 10.7759/cureus.57095

**Published:** 2024-03-27

**Authors:** Stalin R, Angusubalakshmi R, Priya P

**Affiliations:** 1 Community Medicine, Saveetha Medical College and Hospital, Saveetha Institute of Medical and Technical Sciences (SIMATS), Chennai, IND

**Keywords:** monitoring, quality improvement, non-communicable disease, plan-do-study-act, root cause analysis

## Abstract

Introduction

Non-communicable diseases (NCDs) present a significant public health challenge globally, and India is deeply affected. With the largest population in the world, India struggles with a high burden of NCDs, encompassing cardiovascular diseases, diabetes, cancer, and chronic respiratory conditions. These ailments contribute substantially to morbidity and mortality, placing a strain on healthcare systems. Despite efforts through public health initiatives, NCD monitoring and management remain deficient, especially at grassroots levels.

Methods

At a sub-district hospital in Tamil Nadu, India, a quality improvement initiative targeted diabetes and hypertension, prevalent NCDs. Utilizing Fishbone analysis and process flow diagrams, we identified gaps in NCD monitoring. Employing the Plan-Do-Study-Act model and reorienting the patient flow, we enhanced NCD monitoring by optimizing patient health record maintenance within the hospital.

Results

Root cause analysis identified a lack of patient record protocols and patient loss of records as key hindrances in NCD monitoring. We revamped patient flow and implemented a robust record-keeping system, boosting access to patient health records. This initiative was embraced by healthcare providers, enhancing NCD management. Leveraging these records, we assessed control rates of diabetes and hypertension patients effectively.

Conclusion

The research underscores the importance of maintaining comprehensive patient health records in healthcare centers for enhancing NCD monitoring. These records serve as valuable tools for healthcare providers, aiding in the monitoring and treatment of patients with diabetes and hypertension. By leveraging these records, healthcare providers can achieve better disease control outcomes, thereby improving the overall management of NCDs.

## Introduction

Non-communicable diseases (NCDs) pose a significant and growing public health challenge worldwide, and India is no exception. With a population of over 1.3 billion people, India grapples with a high burden of NCDs, including diabetes, cancer, cardiovascular diseases, and chronic respiratory conditions [1]. These illnesses are preventable but are not curable and are brought on by a confluence of behavioral, physiological, environmental, and hereditary variables. In contrast to infectious illnesses, NCDs are often chronic and the product of a complex web of interrelated causes. These diseases are responsible for a substantial proportion of morbidity and mortality in India, placing a considerable strain on healthcare systems and resources [2]. NCDs account for over 74% of deaths worldwide and 60% of deaths in India causing the majority of morbidity and mortality. Due to factors including changing lifestyles and urbanization, the nation is seeing an increase in the prevalence of diabetes, cardiovascular illnesses, and chronic respiratory disorders [3].

State-level NCDs, like those in Tamil Nadu, follow the national trend; therefore, addressing these widespread health issues would need focused interventions. The effectiveness of NCD monitoring in these settings is often hampered by a range of systemic challenges and barriers [4]. The state of Tamil Nadu, located in southern India, is no stranger to the NCD burden, with a significant portion of its population affected by these conditions. A methodical investigation technique called root cause analysis (RCA) aims to pinpoint the underlying causes of a problem or issue. RCA in the healthcare industry is a careful investigation of the root causes of any gaps or weaknesses in patient care or system operations. RCA aids in the classification of possible causes and the visual representation of the phases in a process by using tools such as fishbone analysis and process flow diagrams [5]. To put treatments into practice and make adjustments, the Plan-Do-Study-Act (PDSA) paradigm is often used. By addressing the underlying causes, RCA helps healthcare practitioners continuously improve the efficacy, safety, and quality of the treatment they give. This study aims to conduct a comprehensive analysis of the factors contributing to gaps in NCD monitoring in a sub-district hospital in Tamil Nadu, with the ultimate goal of informing targeted interventions to strengthen NCD management at the grassroots level.

## Materials and methods

**Figure 1:**
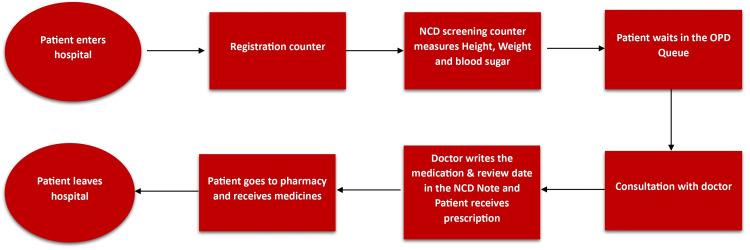
Process flow diagram showing the patient pathway at the sub-district hospital NCD: Non-communicable disease, OPD: Outpatient department.

In response to the pressing need to enhance the monitoring of NCDs at a sub-district hospital in Tamil Nadu, a quality improvement initiative was undertaken during May and June 2023, with a particular focus on diabetes and hypertension. Recognizing the significance of these two prevalent NCDs and their substantial impact on public health, the initiative aimed to identify and address gaps in NCD monitoring practices within the hospital setting. The study setting is a sub-district hospital because of the high NCD prevalence in urban settings. To identify the root cause of the lack of NCD monitoring, we first conducted a baseline assessment and mapped the existing process using a process flow diagram (Figure 1).

**Figure 2:**
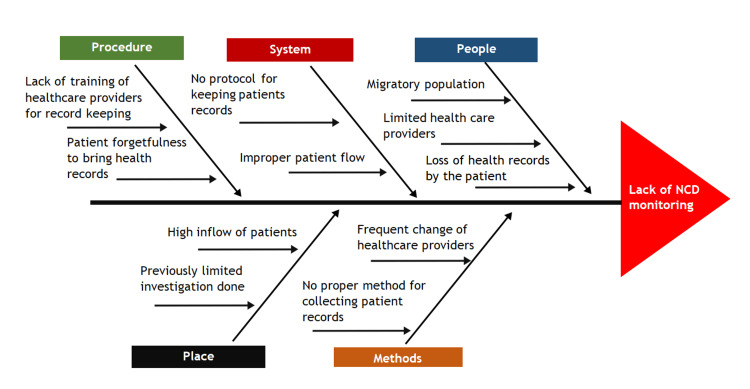
Fishbone analysis diagram Fishbone diagram illustrating the root cause analysis conducted to identify the gaps in Non-communicable disease (NCD) monitoring.

Employing a systematic approach, the initiative utilized fishbone analysis (Figure 2) to conduct a comprehensive assessment of current monitoring processes through brainstorming sessions by the analyzing team, identifying the root causes of inefficiencies and shortcomings [6]. Fishbone analysis, also known as Ishikawa or cause-and-effect analysis, facilitated a structured examination of factors contributing to suboptimal NCD monitoring. By categorizing potential causes into distinct branches such as personnel, processes, equipment, and environment, the analysis provided a holistic view of the underlying issues affecting diabetes and hypertension management within the hospital.

**Figure 3:**
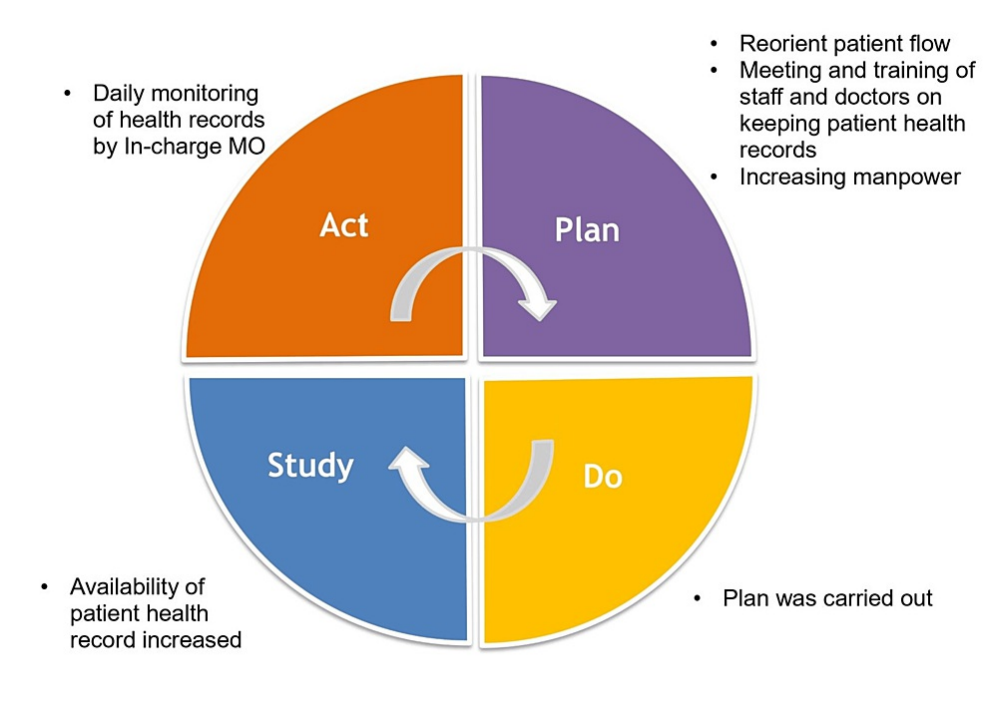
Plan-Do-Study-Act (PDSA) cycle to increase the availability of patient health record MO: Medical officer

In parallel, process flow diagrams were employed to visually map out the sequential steps involved in NCD monitoring, from initial patient assessment to follow-up care. This visual representation facilitated the identification of bottlenecks, redundancies, and opportunities for improvement within the monitoring workflow [7]. By delineating each stage of the process and highlighting points of divergence or inefficiency, the diagrams served as a valuable tool for pinpointing areas ripe for intervention and optimization. Building upon the insights gleaned from the fishbone analysis and process flow diagrams, the initiative embraced the Plan-Do-Study-Act (PDSA) model (Figure 3) as a framework for iterative quality improvement. This systematic approach involved four key phases: planning interventions based on identified gaps (Plan), implementing interventions on a small scale (Do), evaluating their impact through rigorous study and analysis (Study), and iteratively refining and scaling successful interventions (Act).

## Results

The root cause analysis conducted to identify the gaps in NCD monitoring found a lack of standardized protocols and guidelines for NCD monitoring. Within the hospital, variability in screening practices, diagnostic approaches, treatment protocols, and follow-up procedures contributed to inconsistencies and gaps in the delivery of NCD care. Addressing this root cause required the development and implementation of standardized protocols for NCD screening, diagnosis, treatment, and follow-up, ensuring consistency and quality in care delivery across healthcare teams and departments.

Staffing shortages emerged as a significant root cause affecting NCD monitoring practices within the hospital. Insufficient healthcare professionals trained in NCD management were identified through brainstorming sessions which contributed to increased workloads, limited capacity for patient engagement, and challenges in delivering comprehensive care to individuals with chronic conditions. To address this root cause, the initiative focused on four workforce development initiatives, including training programs, recruitment efforts, and workload management strategies designed to optimize staffing levels and enhance the capacity of healthcare teams to deliver high-quality NCD care.

Additionally, infrastructure limitations and resource constraints were identified as root causes impacting NCD monitoring within the hospital. Inadequate access to diagnostic equipment, laboratory facilities, medications, and other essential resources hindered the ability of healthcare providers to accurately diagnose, monitor, and manage NCDs effectively [8]. Furthermore, deficiencies in information systems, data management practices, and technological infrastructure impeded the documentation, retrieval, and utilization of patient health records, undermining the continuity and quality of care. Communication gaps and a lack of interdisciplinary collaboration hindered the continuity, efficiency, and effectiveness of NCD care. The analysis revealed that fragmented systems contributed to delays in referrals, disjointed care pathways, and inconsistencies in treatment approaches, undermining the quality of NCD monitoring services provided to patients [9].

**Figure 4:**
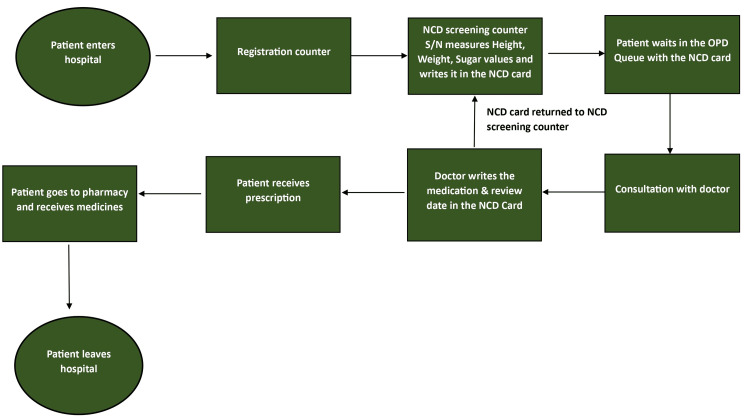
Process flow diagram showing reorientation of the patient flow at the sub-district hospital NCD: Non-communicable disease, S/N: Staff nurse, OPD: Outpatient Department.

To address this root cause, the initiative implemented strategies(Plan-Do-Study-Act cycle and Reoriented patient flow) to improve communication channels, enhance collaboration among healthcare teams, and streamline care coordination processes, ensuring seamless transitions and continuity of care for individuals with NCDs. The root cause analysis conducted as part of the quality improvement initiative provided valuable insights into the systemic challenges and operational deficiencies affecting NCD monitoring practices within the sub-district hospital in Tamil Nadu. By identifying key root causes, the initiative was able to develop targeted interventions aimed at addressing gaps in NCD care delivery and improving patient outcomes [10]. Recognizing the critical importance of accurate and accessible patient health records in facilitating effective NCD management, we embarked on a comprehensive reorientation of the patient flow process (Figure 4) and devised a systematic approach to record-keeping within the hospital [11]. This reorientation ensured that patient health records became an integral part of the care delivery process, rather than an ancillary administrative task. Simultaneously, we devised a system for record-keeping that incorporated standardized protocols, electronic health record (EHR) systems, and staff training to enhance the availability and utilization of patient health records for NCD management [12].

The lack of a standardized protocol for keeping patient records, coupled with the loss of health records by patients, represents significant challenges contributing to the deficiency in NCD monitoring within the sub-district hospital in Tamil Nadu. Patient health records serve as vital documentation that facilitates effective monitoring, management, and continuity of care for individuals with chronic conditions such as hypertension and diabetes [13]. The absence of a standardized protocol for keeping patient records results in variability in documentation practices among healthcare providers, departments, and shifts within the hospital. This lack of uniformity hampers communication and coordination among healthcare teams, hindering the continuity of care and compromising the quality of NCD monitoring services provided to patients. Furthermore, the loss of health records by patients exacerbates the challenges associated with NCD monitoring within the hospital setting [14]. Patients may inadvertently misplace or lose their health records due to factors such as mobility, socioeconomic status, literacy levels, or a lack of awareness about the importance of retaining medical documentation. As a result, healthcare providers may encounter difficulties accessing accurate and up-to-date information about patients' medical histories, treatment plans, medication regimens, and follow-up appointments. This loss of continuity in patient health records undermines the effectiveness of NCD monitoring efforts, as it impedes the ability to track disease progression, assess treatment outcomes, and provide timely interventions.

Investing in training and education programs for healthcare providers and staff on record-keeping best practices, data privacy regulations, and the importance of maintaining complete and updated patient records can enhance awareness and compliance with record-keeping standards.

## Discussion

The root cause analysis conducted as part of the quality improvement initiative to address gaps in NCD monitoring at a sub-district hospital in Tamil Nadu revealed several systemic challenges and operational deficiencies contributing to suboptimal patient care and outcomes. By conducting root cause analysis on medication-related incidents, healthcare professionals can implement strategies to enhance medication safety protocols and reduce errors that may treatment efficacy [15]. Quality improvement principles help us maintain patients' health records in a primary healthcare setting, which can be used for the monitoring of patients with NCDs. India is moving towards digitalization, and a system of a unique health ID for all its citizens has been made under the Digital Health Mission initiative. This is the first step towards creating safer and more efficient digital health records and will go a long way in improving the patient monitoring system [16]. Through a structured examination of the underlying factors influencing NCD monitoring practices, the analysis identified key root causes that informed targeted interventions aimed at improving the quality and effectiveness of NCD care delivery within the hospital setting [17]. Leveraging technology solutions such as electronic health records (EHR) systems, barcode identification, and cloud-based storage platforms can enhance the accessibility, security, and interoperability of patient health records, reducing the risk of loss or damage [18]. Without clear and uniform guidelines outlining the steps and standards for NCD monitoring, healthcare providers may adopt ad-hoc approaches to care delivery, leading to disparities in patient outcomes and missed opportunities for early intervention and disease management [19].

The analysis revealed that staffing shortages were exacerbated by factors such as high patient volumes, turnover rates, and competing priorities within the healthcare system [20]. By employing tools such as fishbone analysis, process flow diagrams, and the PDSA model, the initiative identified opportunities for optimization and implemented targeted interventions to enhance the quality of NCD monitoring [21]. RCA works only at the system level, finding the lapses in the process, and not at the individual level. To examine and determine the required adjustments at a methodical level that can enhance performance and lessen the possibility of a reoccurring sentinel incident, a designated RCA team must be put together. In order to determine the underlying cause of medical errors and enable healthcare institutions to create plans to enhance treatment and avoid errors in the future, the Joint Commission has established a standardized root cause analysis (RCA) process [22]. When used properly, RCA can highlight areas that need to be changed and, in some healthcare situations, can produce testable hypotheses. Since RCA gives attempts to learn from past failures a formal structure, it is worth giving more thought to [23]. Our team's work looked at the same types of RCA solutions as are covered here, and via conversations with safety science specialists and front-line staff, we established a model of solutions that are both sustainable and effective. The study employed the categories established in this project to evaluate the viability and efficacy of various solutions put up by RCA teams. This research demonstrated that the most successful and long-lasting solutions were those that involved changing institutions and technology, denoting the institutions that have proper NCD monitoring and use of appropriate technology for better healthcare, whereas the least successful and long-lasting ones were counseling and disciplinary measures [24]. Many people now utilize root cause analysis as a primary technique for learning from errors and reducing risks. While there have been some advantages, such as greater awareness of flawed procedures and solutions to particular issues, there is a general belief that this strategy is ineffective [25].

The limitation of this study was that it was conducted in an urban sub-district hospital setting, in a single health center, and included monitoring only diabetes and hypertension. By RCA, gaps in the monitoring of the other NCDs can also be addressed, which further helps us strengthen the overall NCD monitoring.

## Conclusions

This quality improvement initiative undertaken at a Tamil Nadu sub-district hospital has significantly improved NCD monitoring and patient care outcomes. Through a comprehensive root cause analysis, we identified the lack of protocol for keeping patient records and the loss of health records by patients as major barriers to effective NCD management within our healthcare facility. In response, we implemented a series of targeted interventions aimed at reorienting the patient flow process and devising a systematic approach to record-keeping. Similarly, gaps in the policies can be identified using RCA, which in turn helps in the better implementation of national health programs.
